# Substrate-Dependent Pro- and Antiarrhythmic Action of Omecamtiv Mecarbil at the Ventricular and Atrial Level: Insights from a Langendorff-Perfused Rabbit Heart Model

**DOI:** 10.1007/s12012-026-10200-0

**Published:** 2026-09-28

**Authors:** Julian Wolfes, Katharina Neff, Felix Wegner, Benjamin Rath, Florian Doldi, Lars Eckardt, Gerrit Frommeyer, Christian Ellermann

**Affiliations:** Department of Cardiology II (Electrophysiology), University Hospital Münster, Albert-Schweitzer-Campus 1, 48149 Münster, Germany

**Keywords:** Langendorff, Omecamtiv mecarbil, Ventricular arrhythmias, Atrial fibrillation

## Abstract

Omecamtiv mecarbil is a cardiac-selective myosin activator and potential heart failure treatment, but its electrophysiological profile remains incompletely characterized. We investigated omecamtiv mecarbil in a Langendorff-perfused rabbit heart model. Forty-eight rabbit hearts were retrogradely perfused; twenty-four in a ventricular and twenty-four in an atrial setup. In the ventricular setup, action potential duration at 90% repolarization (APD_90_), QT interval, and dispersion of repolarization were assessed during pacing at seven cycle lengths. After baseline recordings, Group 1 (*n* = 12) received 1, 5 and 10 µM omecamtiv mecarbil sequentially, while Group 2 (*n* = 12) received 100 µM sotalol followed by 5 µM omecamtiv mecarbil. In the atrial setup, Group 3 (*n* = 12) received 5 and 10 µM omecamtiv mecarbil, and Group 4 (*n* = 12) received isoproterenol/acetylcholine (IsoACh) to increase atrial fibrillation (AF) susceptibility, followed by 5 µM omecamtiv mecarbil. Omecamtiv mecarbil alone shortened ventricular APD_90_ and QT interval, increased dispersion of repolarization, and raised ventricular arrhythmia incidence. Sotalol prolonged APD_90_, QT interval, and dispersion, with a marked rise in VT episodes; additional omecamtiv mecarbil did not further prolong repolarization but increased arrhythmia susceptibility further. In the atrial setup, omecamtiv mecarbil alone prolonged conduction time and increased AF inducibility. Under IsoACh, which shortened atrial APD and refractoriness and increased AF episodes, additional omecamtiv mecarbil slowed atrial conduction and markedly suppressed AF. In summary, omecamtiv mecarbil was proarrhythmic at the ventricular level, particularly under reduced repolarization reserve, but showed antiarrhythmic effects in an IsoACh-induced AF model and proarrhythmic properties in the healthy atrium, indicating a substrate-dependent electrophysiological profile.

## Introduction

Despite considerable progress in the management of chronic heart failure, heart failure with reduced ejection fraction (HFrEF) remains associated with substantial morbidity and mortality [1, 2]. While modern guideline-directed medical therapy has significantly improved survival, drugs with direct positive inotropic action have failed to demonstrate a comparable benefit, particularly in patients with decompensated heart failure or cardiogenic shock; concerns regarding their proarrhythmic potential persist [3–5].

Omecamtiv mecarbil, a first-in-class cardiac myosin activator that enhances myocardial contractility through direct and selective binding to cardiac myosin [6]. Omecamtiv mecarbil has therefore been viewed with considerable expectation. In preclinical and early clinical studies, omecamtiv mecarbil dose-dependently prolonged left ventricular systolic ejection time and improved indices of systolic function, including fractional shortening [7–9]. Rather than acting as a conventional Ca²⁺-dependent inotrope, omecamtiv mecarbil directly modulates cardiac myosin and prolongs systolic contraction without substantially increasing intracellular Ca²⁺ transients. Experimental studies have further demonstrated increased myofilament Ca²⁺ sensitivity and an increased proportion of strongly actin-bound myosin heads, accompanied by slower contraction and relaxation kinetics and, at higher concentrations, no further increase in fractional sarcomere shortening [10, 11]. Thus, the cardiotonic effects of omecamtiv mecarbil appear to result predominantly from altered myosin cross-bridge kinetics, prolonged systolic contraction, and increased myofilament Ca²⁺ sensitivity rather than from an increase in intracellular Ca²⁺ availability or a consistent increase in peak contractile force. Because the contractile gain is achieved without an increase in the cytosolic Ca²⁺ transient, omecamtiv mecarbil has been hypothesized to carry only a low proarrhythmic risk [6]. Although the multicenter GALACTIC-HF trial did not detect a clinically relevant proarrhythmic signal [12, 13], the attenuated benefit observed in patients with concomitant atrial fibrillation in GALACTIC-HF [14] and experimental observations of periodic electromechanical alternans [15] have reopened the discussion on the precise electrophysiological profile of omecamtiv mecarbil.

Against this background, we investigated omecamtiv mecarbil in an established Langendorff model of the isolated rabbit heart, focusing on the effects of omecamtiv mecarbil on ventricular and atrial electrophysiological parameters.

## Methods

The experimental protocol was approved by the local animal welfare office and the responsible state authority (Landesamt für Natur, Umwelt und Verbraucherschutz Nordrhein-Westfalen, file number 81-02.05.50.21.004). The Langendorff whole-heart setup has been described in detail in previous publications from our group [16–18].

In brief, 48 female rabbit hearts (age ≥ 12 weeks; body weight ≥ 2.5 kg) were excised after euthanasia with thiopental and exsanguination. Hearts were aortically cannulated on a Langendorff apparatus and retrogradely perfused with warmed and oxygenated (95% O₂, 5% CO₂) Krebs–Henseleit buffer (NaCl 118 mM, NaHCO₃ 24.88 mM, D-glucose 5.55 mM, KCl 4.70 mM, sodium pyruvate 2 mM, CaCl₂ 1.80 mM, KH₂PO₄ 1.18 mM, MgSO₄ 0.83 mM). Hearts were subsequently allocated to either a ventricular or an atrial experimental setup, depending on the electrophysiological parameters of interest.

### Ventricular Electrophysiology and Arrhythmia Susceptibility

Seven epicardial catheters were placed for monophasic action potential (MAP) recording, complemented by one endocardial catheter positioned in the left ventricle. A pseudo-12-lead surface ECG was recorded using the warming bath surrounding the heart. Mechanical AV nodal ablation was performed prior to the pacing protocol.

Ventricular pacing was performed from an anteroseptal epicardial site on the anterior ventricular wall through an additional pacing catheter at seven cycle lengths between 900 and 300 ms, with simultaneous determination of cycle-length-dependent monophasic action potential duration and QT interval. At each cycle length, pacing was maintained for one minute to allow stabilization, after which the final 16 consecutive beats were recorded for analysis. Subsequently, single short-coupled extrastimuli were applied from the same anteroseptal epicardial pacing site to determine the ventricular effective refractory period (ERP); accordingly, the reported ERP reflects refractoriness at this paced anteroseptal ventricular epicardial site. A burst-pacing protocol consisting of three 50-Hz bursts of 1-s duration was then used to further assess ventricular vulnerability. Hearts were then perfused with hypokalaemic Krebs–Henseleit buffer (K⁺ 1.5 mM) to generate a proarrhythmic hypokalaemic milieu [19]. As myocardial perfusion occurred exclusively via the coronary circulation, this hypokalaemic modification was confined to the coronary perfusate; the surrounding warming bath, which served only to maintain temperature and to provide a conductive medium for the surface ECG, retained a standard, non-hypokalaemic composition.

Action potential duration at 90% repolarization (APD_90_) and the spatial dispersion of repolarization — calculated as the difference between the longest and shortest APD_90_ across the eight monophasic action potentials — served as primary electrophysiological parameters. Post-repolarization refractoriness (PRR) was calculated as the difference between ERP and APD_90_.

### Atrial Electrophysiology and Tachyarrhythmias

Five epicardial catheters were placed on the left and right atrium, with two recording MAPs on each atrium and one MAP for stimulation at the interatrial septum. Hearts were paced at cycle lengths of 350, 250, and 150 ms for one minute each to allow stabilization of the action potential. At each cycle length, 16 consecutive beats were recorded for analysis of atrial action potential duration (aAPD_90_). Interatrial conduction time was evaluated at each cycle length as the interval between the corresponding activation upstrokes in the right- and left-atrial MAP recordings during septal pacing. The atrial effective refractory period (aERP) and atrial post-repolarization refractoriness (aPRR) were determined analogously to the ventricular protocol. Burst pacing consisted of ten 50-Hz bursts of 1-s duration and was used to assess arrhythmia susceptibility.

After determination of baseline electrophysiological parameters, the 48 hearts were allocated to four groups. In group 1, twelve hearts (*n* = 12) were treated sequentially with 1, 5 and 10 µM omecamtiv mecarbil. In group 2, twelve hearts (*n* = 12) were perfused with 100 µM sotalol followed by 5 µM omecamtiv mecarbil.

Because the electrophysiological effects of omecamtiv mecarbil in the ventricular setup were most pronounced at 5 and 10 µM, and because reliable Langendorff perfusion of the atrial preparation could be maintained for a maximum of two sequential drug incubation periods in our experience, the atrial dosing protocol was restricted to these two concentrations. Group 3 (atrial setup, *n* = 12) was perfused with 5 µM omecamtiv mecarbil after baseline recordings. Following a 15-min incubation period, the pacing protocol was repeated. Hearts were subsequently perfused with 10 µM omecamtiv mecarbil and re-evaluated. Each pharmacological intervention was preceded by a 15-min run-in period.

**Fig. 1:**
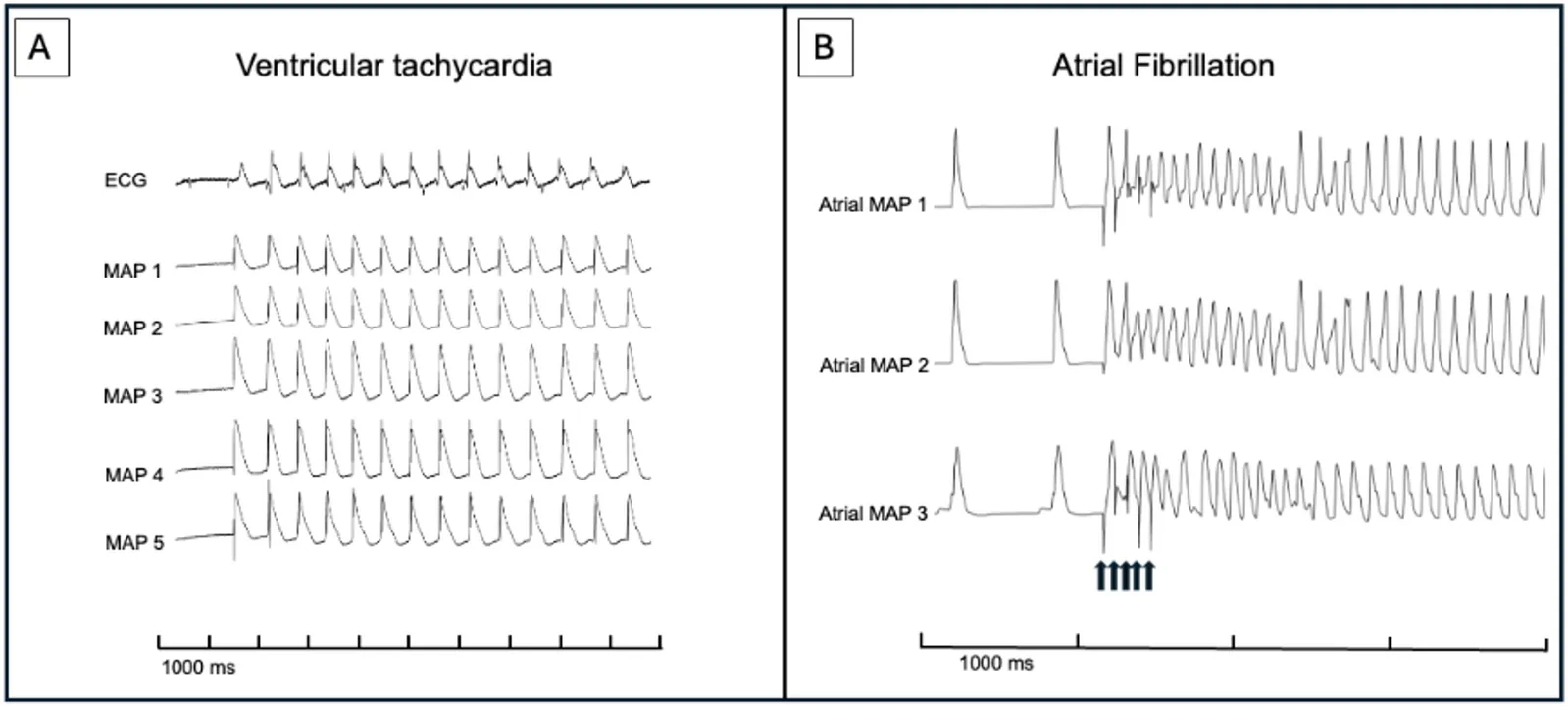
**A** Spontaneous monomorphic VT episode with the corresponding ECG and MAP recordings. **B** Atrial fibrillation induced by burst pacing (arrows)

Group 4 (atrial setup, *n* = 12) employed an arrhythmia-prone substrate. After baseline determination, hearts were perfused with a combination of isoproterenol (1 µM) and acetylcholine (1 µM) to increase AF susceptibility. The combination of both drugs has previously been validated in this [20] and other experimental models [21]. Subsequently, 5 µM omecamtiv mecarbil was added. A representative episode of a monomorphic VT and burst-pacing induced atrial fibrillation with corresponding MAP recordings is shown in Fig. 1.

Electrograms and action potentials were recorded on a multichannel recorder and digitized at 1 kHz with 12-bit resolution. Variables are reported as mean ± standard deviation. Statistical analysis and graphical visualization were performed with GraphPad Prism (version 10). Drug effects on APD_90_, QT interval, spatial dispersion of repolarization, and post-repolarization refractoriness were analyzed using a mixed-effects model. Two-tailed p values < 0.05 were considered statistically significant.

Due to the partially missing measured values at different cycle lengths, a variance analysis in the form of an ANOVA (analysis of variance) with repeated measures was not suitable for the evaluation [22]. The exclusion of further measured values caused by the ANOVA would further reduce the data set and thus also significantly influence the statistical evaluation. Instead, the “mixed model” from the program GraphPad Prism (Version 10) was used as an approximation. This model uses a composite symmetric covariance matrix, taking into account “Restricted Maximum Likelihood”. In a data set without missing values, this model behaves very similarly to an ANOVA with measurement reproduction. However, in the case of missing values, it prevents the loss of the entire measurement series of the variable in question. The Geisser–Greenhouse correction was used in the statistical testing. The Tukey method was used to reduce the accumulation of alpha errors [23].

## Results

### Omecamtiv Mecarbil in the Ventricular Setup

**Fig. 2:**
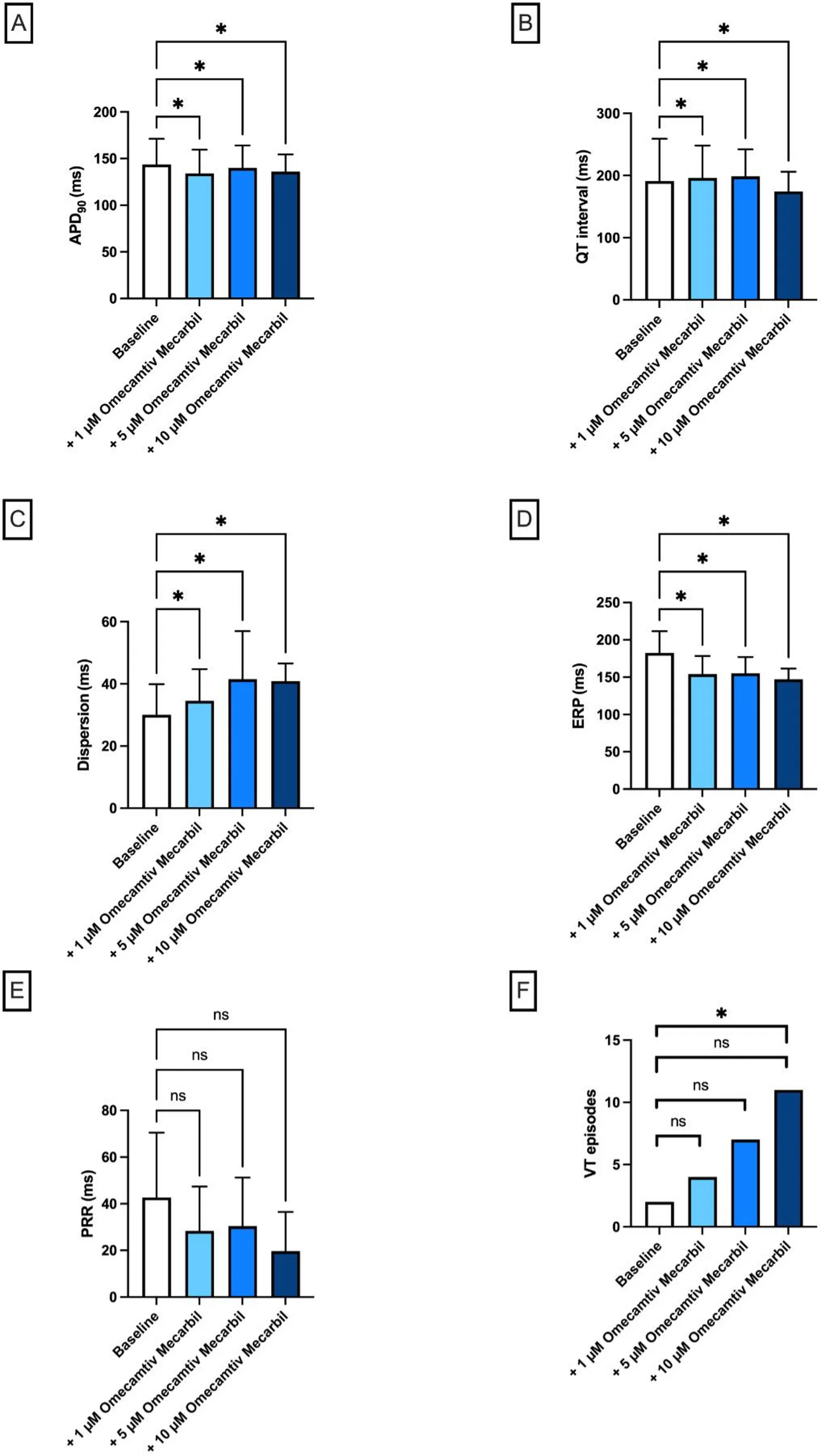
**A** Overall APD_90_ interval and **B** QT interval. Concentration-dependent effect of omecamtiv mecarbil (*n* = 12) on **C** spatial dispersion of repolarization, **D** effective refractory period (ERP), **E** post-repolarization refractoriness (PRR) and **F** number of ventricular tachycardia (VT) episodes. (* = *p* < 0.05), (ns = non-significant)

Perfusion with omecamtiv mecarbil significantly shortened APD_90_ (baseline: 144 ± 28ms; +1µM omecamtiv mecarbil: 134 ± 26ms (*p* < 0.05); +5µM omecamtiv mecarbil: 140 ± 24ms (*p* < 0.05); +10µM omecamtiv mecarbil: 136 ± 18ms (*p* < 0.05)) (Fig. 2). The QT interval displayed a divergent dose-dependent pattern, with prolongation at lower concentrations and shortening at the highest concentration (baseline 191 ± 68 ms; +1 µM 196 ± 52 ms, *p* < 0.05; +5 µM 199 ± 43 ms, *p* < 0.05; +10 µM 175 ± 32 ms, *p* < 0.05). This was accompanied by increased spatial dispersion. The ERP was significantly and the PRR was slightly but not significantly shortened. The incidence of spontaneous ventricular arrhythmias (baseline: 2 episodes in 12 hearts; +1µM omecamtiv mecarbil: 4 episodes in 12 hearts; +5µM omecamtiv mecarbil: 7 episodes in 12 hearts; +10µM omecamtiv mecarbil: 11 episodes in 12 hearts) did only change significantly in the highest doses of omecamtiv mecarbil.

### Omecamtiv Mecarbil in the Long-QT Setup

**Fig. 3:**
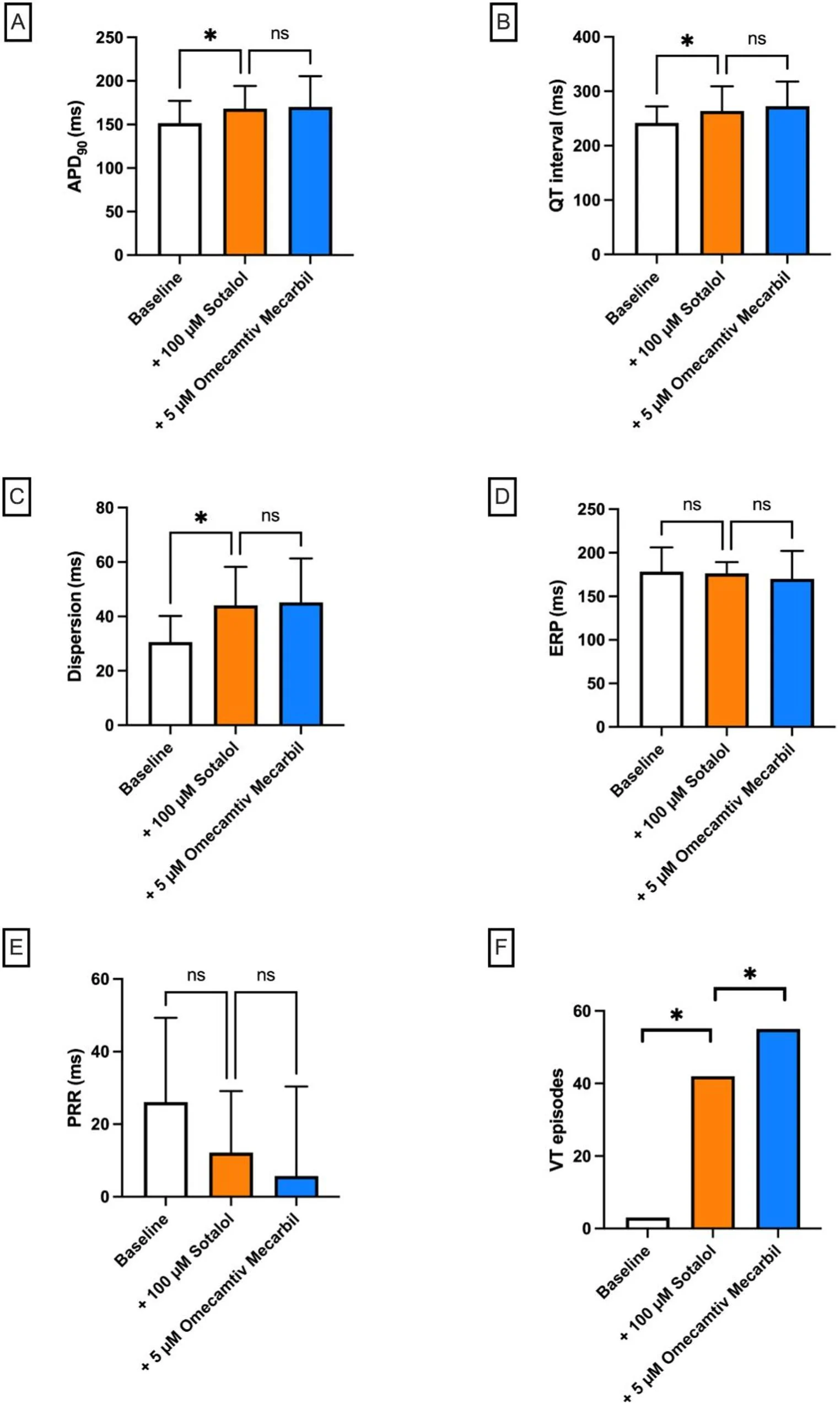
**A** Overall APD_90_ and **B** QT interval. Effect on **C** spatial dispersion of repolarization, **D** effective refractory period (ERP), **E** post-repolarization refractoriness (PRR), and **F** number of ventricular tachycardia (VT) episodes. (* = *p* < 0.05), (ns = non-significant)

Sotalol was employed to induce a pharmacological LQT2 syndrome (Fig. 3). The perfusion with sotalol caused a significant prolongation of the APD_90_ (baseline: 152 ± 25ms; +100µM sotalol: 168 ± 26ms (*p* < 0.05)) and QT interval (baseline: 242 ± 30ms; +100µM sotalol: 264 ± 46ms (*p* < 0.05)). The additional perfusion with omecamtiv mecarbil did not significantly alter both APD_90_ (170 ± 35ms (p = ns)) and QT interval (272 ± 45ms (p = ns)). These observations were paralleled by a significant increase in spatial dispersion of repolarization under sotalol without significant changes under additional perfusion with omecamtiv mecarbil, while ERP and PRR were not significantly altered under both perfusions. The arrhythmia susceptibility was significantly increased under sotalol and was further increased under omecamtiv mecarbil (baseline: 3 episodes in 12 hearts; + 100 µM Sotalol: 42 episodes in 12 hearts (*p* < 0.05); + 5µM omecamtiv mecarbil: 55 episodes in 12 hearts (*p* < 0.05)).

### Omecamtiv Mecarbil on Atrial Electrophysiology

**Fig. 4:**
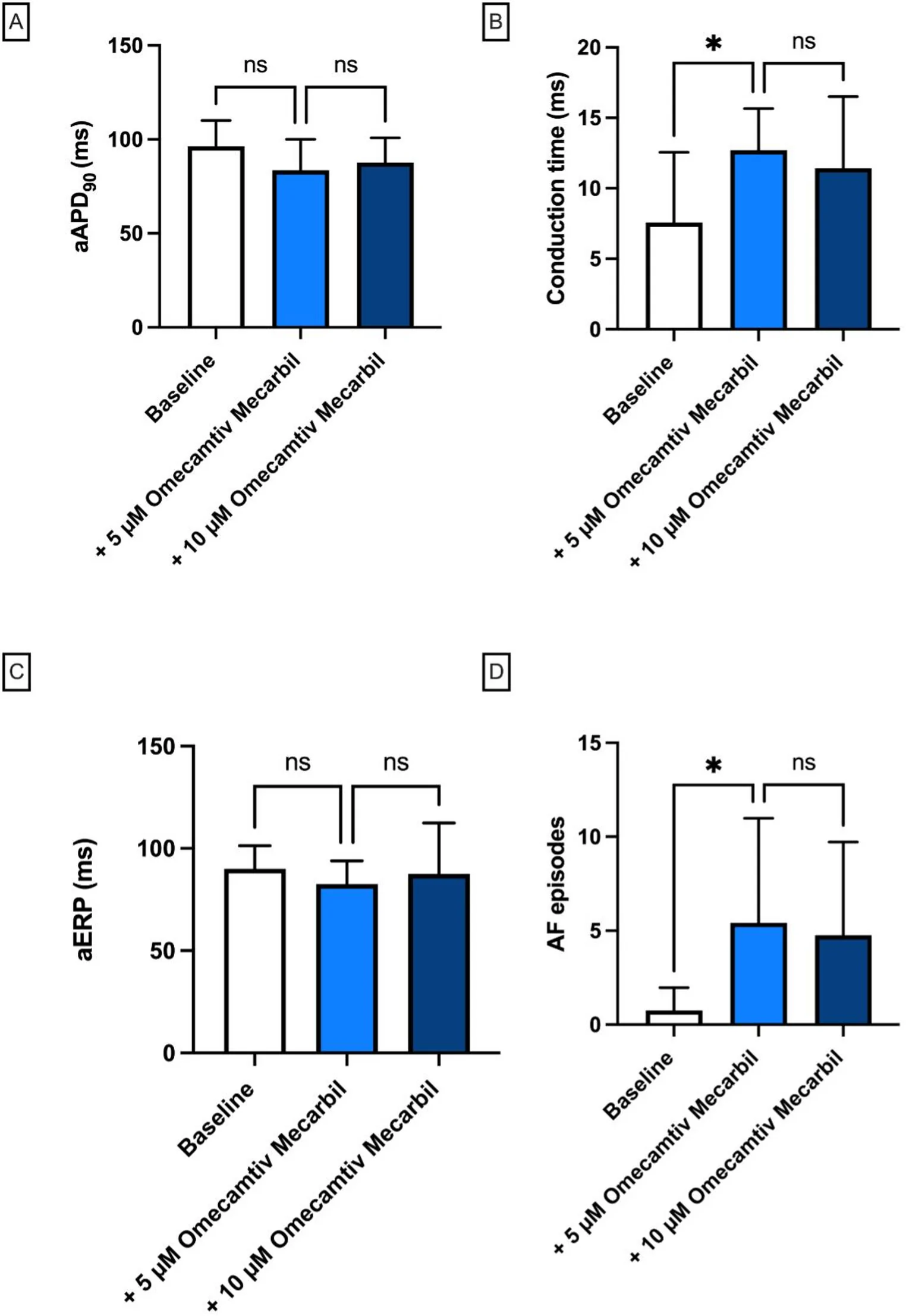
Effect of 5 µM omecamtiv mecarbil and 10µM omecamtiv mecarbil on **A** aAPD_90_, **B** conduction time, **C** atrial effective refractory period (aERP), **D** atrial fibrillation (AF) episodes (* = *p* < 0.05, ns = non-significant)

The perfusion with omecamtiv mecarbil did not significantly alter the aAPD_90_ in the perfused atria (Fig. 4) (baseline: 96 ± 14ms; + 5µM omecamtiv mecarbil: 84 ± 17ms (*p* = ns); +10µM omecamtiv mecarbil: 88 ± 13 ms (*p* = ns)). The atrial conduction time was significantly prolonged under perfusion with omecamtiv mecarbil while the aERP was not significantly altered under both perfusions. The incidence of AF episodes was significantly increased under + 5µM omecamtiv mecarbil and not significantly altered under + 10µM omecamtiv mecarbil.

### Omecamtiv Mecarbil in an Atrial Fibrillation Setup

**Fig. 5:**
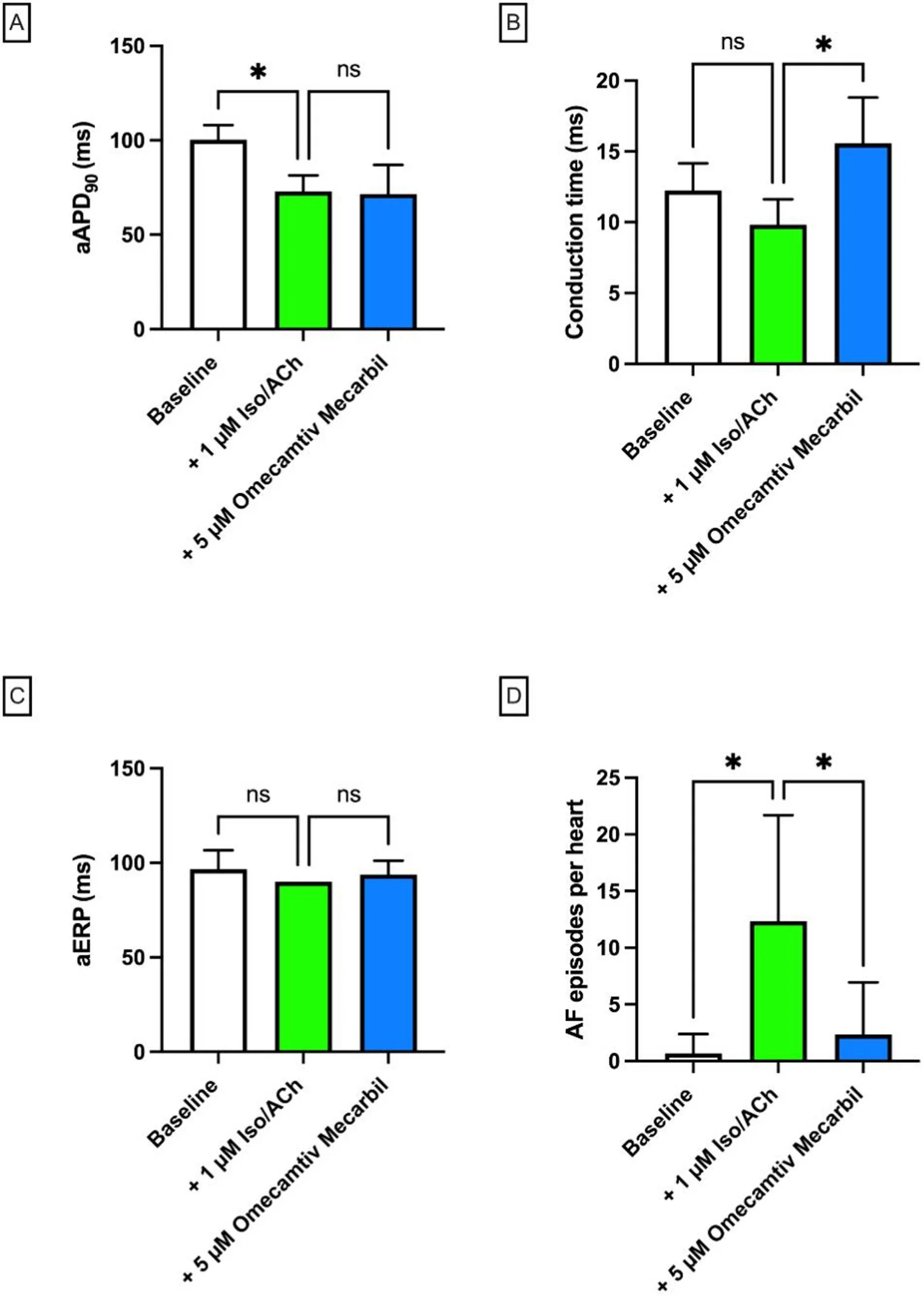
Effect of 1 µM isoproterenol and acetylcholine (IsoACh) and 5µM omecamtiv mecarbil on **A** aAPD_90_, **B** conduction time, **C** atrial effective refractory period (aERP), **D** atrial fibrillation (AF) episodes (* = *p* < 0.05, ns = non-significant)

**Fig. 6:**
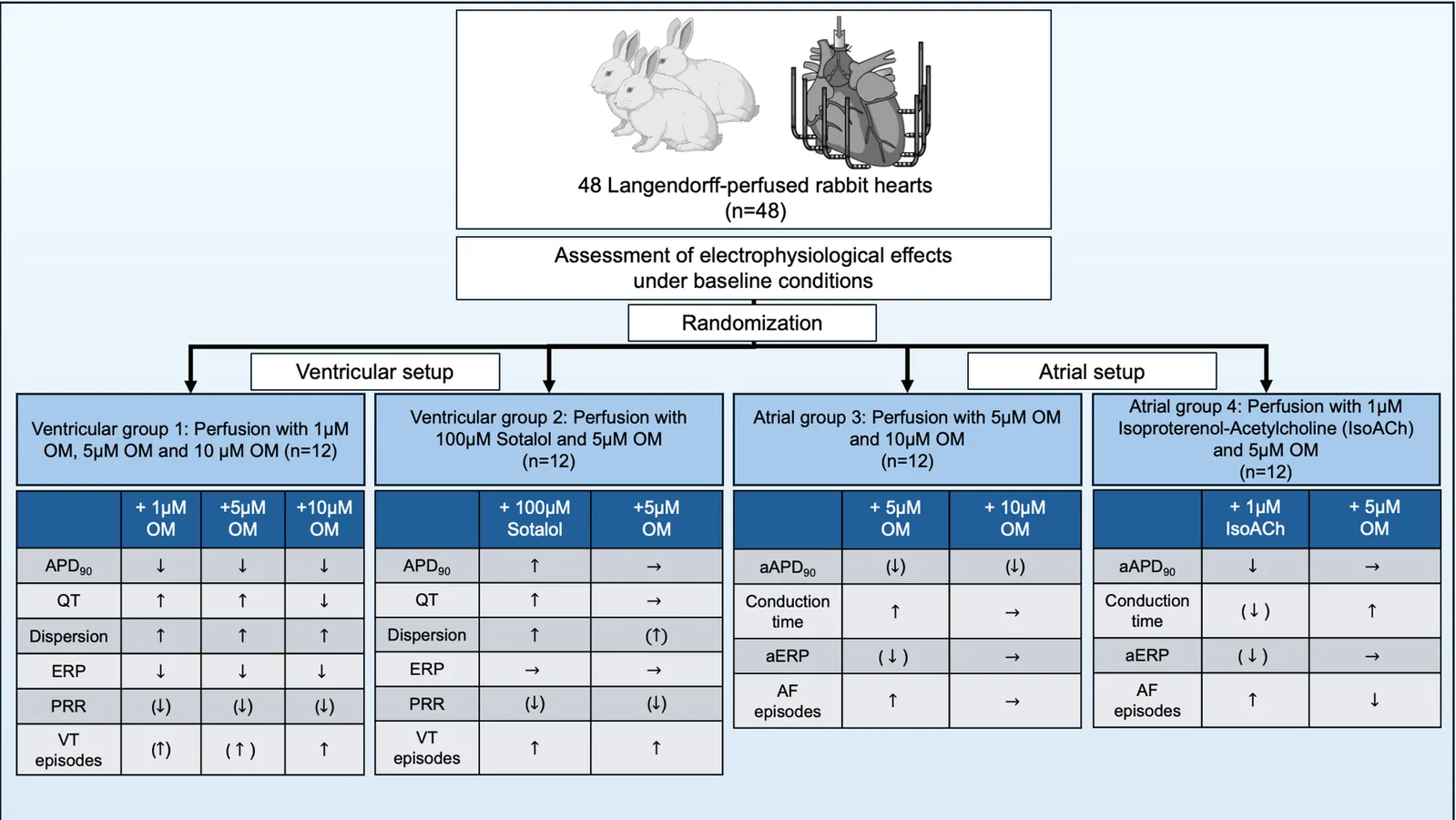
Central illustration of the effects of omecamtiv mecarbil on the cardiac electrophysiology in Langendorff-perfused rabbit hearts. Arrows in brackets indicate effects not reaching the threshold for statistical significance. *APD*_90_ action potential duration at 90% of repolarization, *aAPD*_90_ atrial action potential duration at 90% of repolarization, *ERP* effective refractory period, *aERP* atrial effective refractory period, *PRR* post-repolarization refractoriness, *VT* ventricular tachycardia, *AF* atrial fibrillation, *IsoACh* isoproterenol and acetylcholine, *OM* omecamtiv mecarbil

Perfusion with 1µM isoproterenol and 1µM acetylcholine (IsoACh) was employed to raise susceptibility for atrial fibrillation. Perfusion with IsoACh led to a significant shortening of aAPD_90_ (baseline: 100 ± 8ms; + 1µM IsoACh: 73 ± 9ms (*p* < 0.05)). The additional perfusion with omecamtiv mecarbil did not significantly alter the aAPD_90_ (+ 5µM omecamtiv mecarbil: 72 ± 15ms (p = ns)). The conduction time was significantly shortened under IsoACh and was significantly prolonged beyond baseline under omecamtiv mecarbil. The aERP was slightly but not significantly shortened under IsoACh and slightly but not significantly under omecamtiv mecarbil. The incidence of AF episodes was significantly increased under IsoACh and AF susceptibility was significantly suppressed by omecamtiv mecarbil (Figs. 5 and 6).

## Discussion

### Effects of Omecamtiv Mecarbil on Ventricular Electrophysiology

Increasing concentrations of omecamtiv mecarbil produced a significant, dose-dependent reduction of APD_90_. These findings are in line with previous observations of Szentandrássy et al. [24], who demonstrated APD shortening at 10 and 100 µM omecamtiv mecarbil in canine ventricular myocytes, and of Qu et al. [25], who reported action potential shortening in the canine Purkinje fiber assay. Mechanistically, Szentandrássy and colleagues attributed this effect to inhibition of I_to_ and L-type Ca^2+^ current [24], whereas Qu et al. did not observe relevant inhibition of Ca_v1.2_ [25] and proposed mechanoelectrical feedback secondary to altered contractile properties of the cardiomyocyte as an alternative explanation. The cellular mechanism underlying APD shortening therefore remains incompletely defined.

The dose-dependent divergence between APD_90_ and QT interval — with QT prolongation at 1 and 5 µM but QT shortening at 10 µM omecamtiv mecarbil — may at first appear counterintuitive. However, the surface QT interval reflects not only action potential duration but also myocardial conduction velocity and the spatial dispersion of repolarization, the latter of which is significantly augmented by omecamtiv mecarbil in our experiments. The eventual QT shortening at 10 µM is consistent with data from Qu et al., who also studied perfused rabbit hearts [25] and can be explained by the combined effect of pronounced APD_90_ shortening and only a modest increase in dispersion. Such divergent effects on QT interval and APD_90_ have previously been described by our group [26]. Since omecamtiv mecarbil slowed conduction in the atrial preparation, a comparable effect on ventricular conduction could, in principle, also contribute to the QT prolongation observed at 1 and 5 µM. However, ventricular conduction time and QRS duration were not prospectively quantified in the present protocol; therefore, such an effect cannot be confirmed or excluded from our data.

The trend toward more ventricular tachycardia (VT) episodes at 1 and 5 µM, along with the significant increase at 10 µM, contradicts the “low proarrhythmic risk” attributed to omecamtiv mecarbil by Qu et al. [25] and underscores the value of perfused whole-heart models. Our observations are also concordant with those of Fülöp et al., who described periodic electromechanical alternans — a recognized precursor of ventricular arrhythmias — in 77% of rats receiving doses ≥ 600 µg/kg of omecamtiv mecarbil [15].

A plausible electrophysiological correlate of increased ventricular arrhythmia susceptibility under omecamtiv mecarbil, in the absence of action potential prolongation, can be derived from our data: the central findings are increased spatial dispersion of repolarization and a trend toward shortened PRR. This is consistent with the well-established observation that the QT interval is not a reliable predictor of ventricular proarrhythmia [27–29], whereas measures of dispersion and refractory parameters such as PRR provide more reliable markers.

### Effects of Omecamtiv Mecarbil in the LQT2 Model

As expected, sotalol perfusion significantly prolonged APD_90_, QT interval, and spatial dispersion, and increased the number of ventricular arrhythmic episodes. In this milieu, additional perfusion with 5 µM omecamtiv mecarbil did not further prolong APD_90_, QT interval, or dispersion, but produced a marked, although statistically non-significant, shortening of PRR. This was paralleled by a clear increase in the number of VT episodes. Compared with the absolute number of VT episodes observed under 10 µM omecamtiv mecarbil in group 1, the proarrhythmic potential under sotalol pre-treatment was substantially higher. These data suggest that omecamtiv mecarbil exerts an additive proarrhythmic effect, particularly in the setting of reduced repolarization reserve. A plausible electrophysiological correlate is the further — although not statistically significant — reduction of PRR, which compromises protection against early afterdepolarizations under conditions of QT prolongation.

Our model shows a ventricular proarrhythmic signal, in contrast to the lack of such a signal in GALACTIC-HF [12] and its dedicated subanalysis [13]. Possible reasons include (I) the somewhat higher drug concentrations used in our study compared to the GALACTIC-HF target peak plasma level of up to 2.49 µM [12]; (II) the absence of antiarrhythmic co-medications — especially β-blockers, which most GALACTIC-HF patients received; and (III) the use of healthy rabbit hearts in our study, unlike the failing hearts of clinical trial participants. However, the increased proarrhythmic risk observed during sotalol-induced reduction of repolarization reserve warrants particular attention, especially regarding heart failure patients on QT-prolonging drugs and in conditions like myocardial ischemia with reduced repolarization reserve [30]. An additional methodological difference is the ventricular activation sequence. In our ventricular experiments, AV nodal ablation followed by epicardial ventricular pacing was used to control cycle length, thereby bypassing physiological activation through the specialized His–Purkinje system and producing a non-physiological ventricular activation sequence. In GALACTIC-HF, by contrast, ventricular activation in patients with sinus or atrial rhythm generally occurred through the native conduction system unless ventricular pacing was present. Differences in activation sequence and ventricular synchrony may influence depolarization, repolarization, and mechanoelectrical feedback and could therefore have contributed to the discrepant proarrhythmic findings between the experimental and clinical settings. This explanation remains speculative and cannot be separated from the other model differences discussed above.

### Effects of Omecamtiv Mecarbil on Atrial Electrophysiology

Atrial perfusion with increasing omecamtiv mecarbil concentrations resulted in a slight, though not statistically significant, decrease in aAPD_90_ and atrial refractory period. Atrial conduction time was notably extended, indicating a reduction in atrial conduction velocity, which was associated with a significant rise in AF episodes. This may be explained by Allessie’s classical wavelength-of-reentry theory, where slowed conduction helps stabilize reentrant circuits [31]. Interestingly, the conduction slowing occurred without a corresponding prolongation of the effective refractory period, unlike typical sodium-channel-blocking (class I) antiarrhythmic drugs [32]. Based on Rayo Abella et al.‘s findings of prolonged contractile relaxation in human atrial tissue under omecamtiv mecarbil [33], a mechanoelectrical modulation of atrial electrophysiology might be involved, although this cannot be directly confirmed in the current model.

### Effects of Omecamtiv Mecarbil in the Atrial Fibrillation Model

As anticipated, perfusion with isoproterenol and acetylcholine caused a reduction in atrial action potential duration, conduction time, and refractory period, leading to a significant increase in AF episodes. The incidence of AF was substantially higher than under omecamtiv mecarbil alone in group 3. The combination of isoproterenol and acetylcholine creates a well- known proarrhythmic substrate in isolated perfused hearts: acetylcholine- mediated I_KACh_ activation shortens aAPD and aERP, promoting spatial repolarization heterogeneity, and provides a substrate for short- wavelength reentry, while β- adrenergic activation by isoproterenol enhances cAMP- dependent I_CaL_ and causes Ca^2+^ overload, initiating triggered activity [34, 35].

In this context, omecamtiv mecarbil again significantly prolonged atrial conduction time and decreased conduction velocity, but notably, this was associated with a substantial reduction in AF episodes. Therefore, slowing atrial conduction appears to have substrate- dependent, contrasting effects on atrial arrhythmia susceptibility. This seeming paradox can be explained by the fact that, under isoproterenol and acetylcholine, AF episodes tend to be more complex and disorganized [36], with chaotic, multiple-wavelet AF, in contrast to organized macro-reentrant AF, often terminating when conduction velocity is further reduced [37, 38]. Thus, whether omecamtiv mecarbil exerts pro- or antiarrhythmic effects at the atrial level seems to depend on the specific atrial electrophysiological substrate.

In a subanalysis of GALACTIC-HF by Solomon et al. [14], no increase in new-onset atrial fibrillation was observed with omecamtiv mecarbil (3.1 vs. 3.7 episodes per 100 patient- years in the omecamtiv mecarbil and placebo groups, respectively; *p* = 0.11). This supports the hypothesis that the heart failure population, characterized by sympathetic overdrive and atrial remodeling, generally has an atrial substrate similar to the IsoACh group in this study. It should also be noted that AF patients on digoxin did not benefit from omecamtiv mecarbil, although the underlying mechanisms are not fully understood [14].

### Limitations

Although the Langendorff model of the isolated rabbit heart is a well-established platform for the investigation of electrophysiological mechanisms [39], translation to the human heart is inherently limited. In particular, the present study used exclusively healthy rabbit hearts, whereas omecamtiv mecarbil is intended primarily for patients with reduced left ventricular function.

Furthermore, the model does not allow direct cellular electrophysiological recordings or assessment of transcriptional effects, owing to the temporal dissociation between drug perfusion and electrophysiological recording. The drug concentrations used in the present study were partly above the clinical target plasma levels of up to 2.49 µM reported in GALACTIC-HF [12], although they remain within the range of hypothetically achievable concentrations.

Finally, the present model was designed to characterize electrophysiological rather than mechanical parameters; direct measurements of contractile force, time to peak tension, or relaxation kinetics were not performed, and no chronotropic endpoints were assessed. Prior work in isolated cardiomyocytes and preclinical/clinical studies indicates that omecamtiv mecarbil increases myofilament calcium sensitivity and prolongs systolic ejection time without a proportional increase in maximal Ca2+-activated force or a marked chronotropic effect [7–11]. Whether the associated slowing of cross-bridge kinetics and prolongation of the systolic ejection time contribute to the proarrhythmic phenotype observed here, and how pharmacological modulation of the L-type Ca2 + current (e.g., with nifedipine) would modulate this interaction, could not be addressed in the present electrophysiological setup, but represents an important direction for future mechanistic studies combining force and action potential recordings. In addition, QRS duration and ventricular conduction velocity were not systematically quantified. Therefore, a potential omecamtiv mecarbil-induced slowing of ventricular conduction, and its possible contribution to the observed QT changes, cannot be assessed from the present data.

### Clinical Perspective

These experiments uncovered a complex electrophysiological profile of omecamtiv mecarbil. Notably, the significant ventricular proarrhythmic effect seen in the LQT2 model calls for careful attention in patients taking QT-prolonging co-medications. The atrial profile is particularly interesting, as omecamtiv mecarbil seems to have antiarrhythmic effects in patients predisposed to AF, yet it exhibits proarrhythmic properties in healthy atrial tissue.

## Data Availability

The data supporting the findings of this study are available on request from the corresponding author.

## Abbreviations

AF: Atrial fibrillation
APD_90_: Action potential duration at 90% of repolarization
aAPD_90_: Atrial action potential duration at 90% of repolarization
ECG: Electrocardiogram
ERP: Effective refractory period
aERP: Atrial effective refractory period
HFrEF: Heart failure with reduced ejection fraction
IsoACh: Isoproterenol and acetylcholine
LQT2: Long QT syndrome type 2
OM: Omecamtiv mecarbil
PRR: Post-repolarization refractoriness
aPRR: Atrial post-repolarization refractoriness
VT: Ventricular tachycardia
